# Research Review: A review of the past decade of family and genomic studies on adolescent mental health

**DOI:** 10.1111/jcpp.14099

**Published:** 2024-12-19

**Authors:** Geneviève Morneau‐Vaillancourt, Elisavet Palaiologou, Tinca J.C. Polderman, Thalia C. Eley

**Affiliations:** ^1^ Social, Genetic & Developmental Psychiatry Centre Institute of Psychiatry, Psychology & Neuroscience, King's College London London UK; ^2^ Department of Clinical Developmental Psychology Vrije Universiteit Amsterdam The Netherlands; ^3^ Department of Child and Adolescent Psychiatry & Social Care Amsterdam UMC Amsterdam The Netherlands; ^4^ National Institute for Health Research (NIHR) Biomedical Research Centre, South London and Maudsley Hospital London UK

**Keywords:** Adolescence, psychopathology, behavioural genetics, genomics

## Abstract

**Background:**

Mental health problems and traits capturing psychopathology are common and often begin during adolescence. Decades of twin studies indicate that genetic factors explain around 50% of individual differences in adolescent psychopathology. In recent years, significant advances, particularly in genomics, have moved this work towards more translational findings.

**Methods:**

This review provides an overview of the past decade of genetically sensitive studies on adolescent development, covering both family and genomic studies in adolescents aged 10–24 years. We focus on five research themes: (1) co‐occurrence or comorbidity between psychopathologies, (2) stability and change over time, (3) intergenerational transmission, (4) gene–environment interplay, and (5) psychological treatment outcomes.

**Results:**

First, research shows that much of the co‐occurrence of psychopathologies in adolescence is explained by genetic factors, with widespread pleiotropic influences on many traits. Second, stability in psychopathology across adolescence is largely explained by persistent genetic influences, whereas change is explained by emerging genetic and environmental influences. Third, contemporary twin‐family studies suggest that different co‐occurring genetic and environmental mechanisms may account for the intergenerational transmission of psychopathology, with some differences across psychopathologies. Fourth, genetic influences on adolescent psychopathology are correlated with a wide range of environmental exposures. However, the extent to which genetic factors interact with the environment remains unclear, as findings from both twin and genomic studies are inconsistent. Finally, a few studies suggest that genetic factors may play a role in psychological treatment response, but these findings have not yet been replicated.

**Conclusions:**

Genetically sensitive research on adolescent psychopathology has progressed significantly in the past decade, with family and twin findings starting to be replicated at the genomic level. However, important gaps remain in the literature, and we conclude by providing suggestions of research questions that still need to be addressed.

## Introduction

Mental health problems, hereafter referred to as psychopathology, are common and often begin during adolescence (Caspi et al., 2020). Between 25% and 50% of youths experience at least one psychiatric disorder during adolescence, and approximately 60% of psychiatric disorders emerge before the age of 25 years (Auerbach et al., 2018; Caspi et al., 2020; Merikangas et al., 2010; Silva et al., 2020; Solmi et al., 2022). Psychopathology is associated with considerable personal and economic burden, disrupting both education and employment (Whiteford et al., 2013). As such, extensive research has been conducted on the factors underlying psychopathology in adolescence. Decades of twin studies indicate that genetic factors account for approximately 50% of individual differences in psychopathology (Polderman et al., 2015). Genetically sensitive studies have therefore provided crucial insights into the mechanisms—both genetic and environmental—underlying the onset, persistence, and remission of adolescent psychopathology.

**Figure 1:**
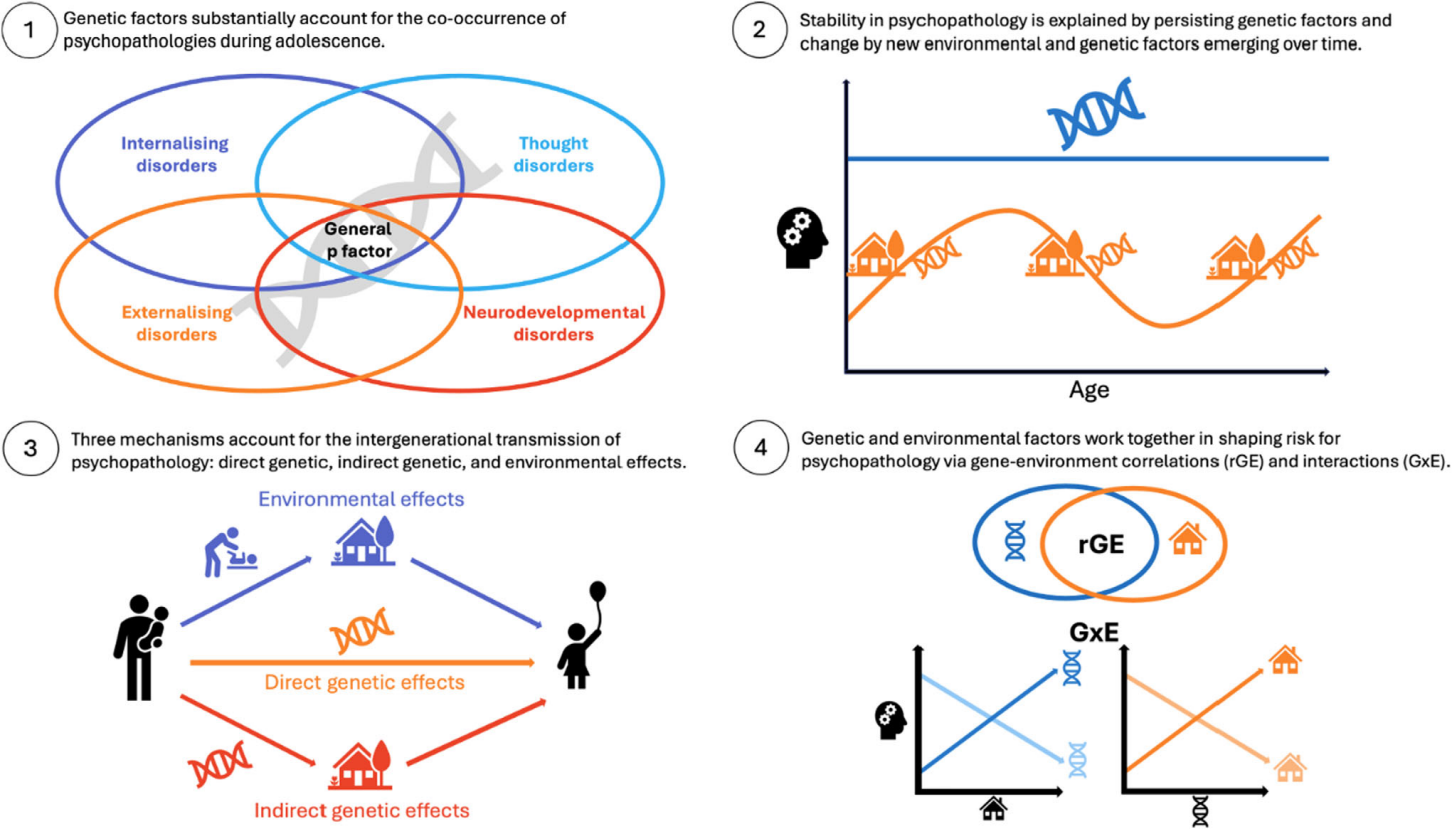
Main findings on the genetics of adolescent psychopathology

Here we provide an overview of findings from the past 10 years of research in this area (for a review of earlier findings, see Plomin, DeFries, Knopik, & Neiderhiser, 2016). We cover five broad research themes, as indicated in Figure 1: (1) co‐occurrence or comorbidity between psychopathologies, (2) stability and change over time, (3) intergenerational transmission, (4) gene–environment interplay, and (5) psychological treatment outcomes. We conceptualise adolescence as ranging from 10 to 24 years old (Sawyer, Azzopardi, Wickremarathne, & Patton, 2018). The brain is still developing over this period and young people in their early twenties often experience significant social instability that delays their transition into adulthood (Arnett, Žukauskienė, & Sugimura, 2014; Huttenlocher, 1990; Pfefferbaum et al., 1994; Sawyer et al., 2018). We do not cover studies using linkage associations and candidate genes, as these methods are ill‐suited to the investigation of complex polygenic traits like psychopathologies (for a discussion, see Andreassen, Hindley, Frei, & Smeland, 2023). Finally, we do not provide descriptions of existing designs and methods and refer readers to relevant reviews instead (see Jami, Hammerschlag, Bartels, & Middeldorp, 2021; McAdams, Cheesman, & Ahmadzadeh, 2023; Willoughby, Polderman, & Boutwell, 2023).

## The importance of genetically sensitive studies

Family‐based methods like twin studies have been conducted for more than 50 years, and they remain some of the most powerful designs to identify genetic, but also environmental influences on psychopathology. Many existing twin studies, some of which launched more than 25 years ago, have rich longitudinal data and provide unique opportunities to examine how genetic and environmental influences unfold across development (for a description of twin registers around the world, see Willoughby et al., 2023). In recent years, genomic studies have complemented twin studies by shedding light on the role of specific genetic variants, enabling prediction and providing insights into the core biological mechanisms involved in psychopathology (Smoller et al., 2019). Researchers are now combining family‐based and genomic methods, as many participants from family studies are starting to have their own children (e.g. Ahmadzadeh et al., 2019). New research questions are being addressed, such as the identification of mechanisms underlying the intergenerational transmission of psychopathology (Cheesman, Ayorech, Eilertsen, & Ystrom, 2023; McAdams et al., 2023). Contemporary, genetically sensitive studies can thus provide crucial insights into the development of psychopathology in adolescents.

## Co‐occurrence of psychopathologies in adolescence

Many adolescents experience more than one psychopathology simultaneously or over time (Caspi et al., 2020). About 40% of adolescents living with psychopathology will be diagnosed with another psychiatric disorder at some point during adolescence (Merikangas et al., 2010). Researchers have proposed different models to account for this co‐occurrence, including the Research Domain Criteria (RDoc; Insel et al., 2010), general psychopathology or p factor model (Caspi et al., 2014; Lahey et al., 2012), and the Hierarchical Taxonomy of Psychopathology (HiTOP; Kotov et al., 2017). These models have their own particularities (see Table 1), but collectively suggest that psychopathologies cluster under broad quantitative dimensions rather than separate categorical diagnoses.

**Table 1:** Key definitions

*Common genetic variants*: Units of DNA that vary across individuals, typically observed in at least 1% of the population, also called single nucleotide polymorphisms or SNPs
*Direct genetic effects*: When an individual's genotype directly affects their observable characteristics (phenotype). For example, common genetic variants that directly influence the development of psychopathology
*Family studies*: Studies that exploit the level of genetic resemblance between family members to assess the extent to which genetic and environmental factors contribute to variability in a trait; for example, adoption, twin, and intergenerational studies
*Gene–environment correlation (rGE)*: When genetic factors increase or decrease the likelihood of experiencing certain environments (Plomin, 2014). There are three types of rGE: passive, evocative, and active. Passive rGE begins from birth and is analogous to genetic nurture (see below), when the genes that parents transmit to their children are correlated with the environment provided by the parents, thereby creating a correlation between children's genotypes and their environment. Evocative rGE also begins early in life and captures instances when an individual's genotype evokes a response in others, for example an adolescent's genotype affecting the way a parent responds to them. Active rGE comes on line latest in development and is seen when an individual's genotype influences their propensity to select environments, for example an adolescent's genotype affecting their likelihood of affiliating with delinquent peers
*Gene–environment interaction (GxE)*: When the effect of the environment on a trait depends on genetic factors or vice versa (Dick, 2011). For example, the impact of exposure to social support or trauma on mental health may depend on an individual's genotype, or the heritability of psychopathology may vary depending on the environmental context
*Genetic nurture*: When *indirect* genetic effects are observed within families. For example, when a parent's genotype influences their provision of social support, for example creating a positive home environment that in turn positively affects a child's mental health
*Genome‐wide association study (GWAS)*: Observational studies, usually including thousands of participants, that examine associations between hundreds of thousands of common genetic variants (SNPs) across the genome and a specific trait (e.g. major depressive disorder)
*Genomic Structural Equation Modelling (Genomic‐SEM)*: Statistical technique integrating genomic data with structural equation modelling (SEM) used to examine the genetic architecture and shared genetic influences between multiple traits (Grotzinger et al., 2019)
*Genomic studies*: Studies relying on a broad range of techniques using genome‐wide genetic data to examine the extent to which genetic variants explain phenotypic variations, for example, GWAS, polygenic score prediction, genomic‐SEM, and Mendelian randomisation
*Heritability*: Estimate representing the extent to which variation in an observable trait (phenotypic differences between individuals) is explained by genetic variation in a population. There are different ways of estimating heritability, for example using twin versus genome‐wide association study data
*Hierarchical Taxonomy of Psychopathology (HiTOP)*: A framework organising psychopathologies along continua or dimensions into a hierarchical structure including several levels: specific symptoms, traits, syndromes or disorders (e.g. panic disorder), slightly broader subfactors (e.g. fear), broad spectra (e.g. internalising), and very broad superspectra (e.g. general psychopathology factor; Kotov et al., 2017)
*Indirect genetic effects*: When one individual's genotype affects another person's phenotype through environmental mechanisms. For example, an adolescent's genotype influencing peers' behaviours through social processes. Genetic nurture is also an indirect genetic effect
*P factor model*: Also known as the general psychopathology factor model, a framework proposing that a single, overarching factor accounts for a wide range of psychopathologies (Caspi et al., 2014; Lahey et al., 2012)
*Polygenic scores (PGS)*: An individual‐specific score representing genetic susceptibility to a specific phenotypic trait (e.g. major depressive disorder). Polygenic scores are constructed by aggregating individual SNPs, weighted according to their effect sizes estimated within a specific GWAS
*Research Domain Criteria*: A dimensional framework incorporating neuroscience and genetic findings that conceptualises psychopathology according to six major domains of human neurobehavioural functioning: negative valence, positive valence, cognitive, social processes, arousal or regulatory, and sensorimotor systems (Insel et al., 2010)
*Single nucleotide polymorphisms (SNP)*: Single nucleotide unit on the DNA sequence that can vary across individuals.
*Therapygenetics*: How an individual's genotype may influence their response to psychological treatment, a type of gene–environment interaction (Eley et al., 2012)
*Twin studies*: Studies that examine how twins growing up within the same family resemble one another to estimate how genetic, shared environmental, and non‐shared environmental (also including measurement error) contribute to a trait. This is possible because fraternal (dizygotic) and identical (monozygotic) twins differ in their level of genetic similarity (50% on average for fraternal twins versus 100% for identical twins) and grow up in the same family

Adapted from de Wit and Polderman (2023).

Studies in adolescents consistently identify three core dimensions of psychopathology (Afzali, Sunderland, Carragher, & Conrod, 2018; Carragher et al., 2016; Castellanos‐Ryan et al., 2016; Laceulle, Vollebergh, & Ormel, 2015; Michelini et al., 2019; Noordhof, Krueger, Ormel, Oldehinkel, & Hartman, 2015; Patalay et al., 2015; Pocuca et al., 2023). The first is internalising disorders, which include anxiety, depression, and somatoform problems. The second is externalising, most often including conduct disorder, oppositional defiant disorder, aggression, delinquency and substance use. The third is a general psychopathology or p factor, influencing all disorders. In addition to these three core dimensions, some studies identify other dimensions, namely substance use as a separate dimension (Castellanos‐Ryan et al., 2016; Pocuca et al., 2023), thought disorders including psychotic experiences (Afzali et al., 2018; Carragher et al., 2016), and neurodevelopmental disorders including attention deficit/hyperactivity disorder (ADHD) and autism spectrum disorder (ASD; Michelini et al., 2019; Noordhof et al., 2015). Traits related to ADHD are sometimes classified under the externalising dimension (e.g. Laceulle et al., 2015). However, ADHD and ASD should be considered as a separate dimension as they have their own defining characteristics, including early age of onset, high heritability, and steady developmental course (Thapar, Cooper, & Rutter, 2017).

### Genetic structure of psychopathology

In support of these broad dimensions, twin studies show significant genetic correlations between psychopathologies within and across these dimensions. High genetic correlations are seen within dimensions, for example, between anxiety and depression (Matthews et al., 2016; Waszczuk, Zavos, Gregory, & Eley, 2014), antisocial behaviour and substance use (McAdams, Rowe, Rijsdijk, Maughan, & Eley, 2012), and ADHD and ASD (Taylor et al., 2013). Similarly, twin studies show significant genetic correlations *across* different dimensions, such as between thought and neurodevelopmental disorders (Taylor et al., 2022), externalising and neurodevelopmental disorders (Kuja‐Halkola, Lichtenstein, D'Onofrio, & Larsson, 2015), internalising and externalising disorders (Savage et al., 2015), or thought and internalising disorders (Zavos et al., 2016). Genomic studies have replicated these twin findings by showing significant genetic correlations between psychopathologies, again both within and across dimensions of internalising, externalising, thought, and neurodevelopmental disorders in children, adolescents, and adults (The Brainstorm Consortium et al., 2018; Waldman, Poore, Luningham, & Yang, 2020; for a review of genomic studies in children and adolescents, see Akingbuwa, Hammerschlag, Bartels, & Middeldorp, 2022). Further evidence for a general psychopathology or p factor comes from twin studies showing that the heritability of p in adolescence ranges from 43% to 87% (Allegrini et al., 2020; O'Reilly et al., 2020; Waldman, Poore, van Hulle, Rathouz, & Lahey, 2016). Likewise, genomic studies indicate that the heritability estimated from common genetic variants (or SNP heritability) of p in childhood and adolescence is around 5% (Alnæs et al., 2018; Neumann et al., 2022). The gap between twin (43%–87%) and SNP heritability (5%) estimates is commonly called the missing heritability problem and illustrates a phenomenon that is consistent across genetic studies: twin heritability is usually much larger than SNP heritability, especially for complex traits like psychopathology (Manolio et al., 2009). Explanations for the missing heritability problem include the fact that twin studies may overestimate heritability or that they may additionally capture genetic interactions, as well as contributions from rare genetic variants not included in GWAS (for discussions on missing heritability, see Eichler et al., 2010; Friedman, Banich, & Keller, 2021; Young, 2019). Nonetheless, there is extensive evidence that psychopathologies in adolescence share common genetic factors and that this genetic overlap supports a dimensional model of psychopathology.

In addition, studies have used polygenic scores computed from genome‐wide association studies (GWAS) in adults to examine their association with adolescent psychopathology. Again, findings show significant genetic correlations both within and across dimensions of psychopathology. Polygenic scores for adult psychopathology have been associated with internalising, externalising, thought disorders and the p factor in childhood and adolescence (Akingbuwa et al., 2020; Brikell et al., 2020; Chen et al., 2022; Jansen et al., 2021; Jones et al., 2016; Kutzner, Elam, & Ha, 2023; Kwong et al., 2021; Nivard et al., 2017; Pain et al., 2018; Riglin et al., 2020; Waszczuk et al., 2023). However, polygenic score findings are sometimes inconsistent. For example, some studies show significant associations between a polygenic score for schizophrenia and the p factor in adolescents (Jones et al., 2018; Riglin et al., 2020), whereas others do not (Chen et al., 2022). As such, polygenic score studies support, for the most part, a dimensional model of psychopathology in adolescence. They also show that genetic liabilities for adult psychopathology may be detected earlier in adolescence, providing some evidence of developmental continuity in genetic risks.

Finally, new genomic structural equation modelling (genomic‐SEM) techniques analogous to twin modelling have been developed in recent years (de Hoyos et al., 2024; Grotzinger et al., 2019; Pritikin, Neale, Prom‐Wormley, Clark, & Verhulst, 2021). To date, genomic‐SEM has mostly been used in adult samples (e.g. Grotzinger et al., 2019, 2022), and so far only one study has used this approach in a sample of children and adolescents, to examine the genetic overlap between symptoms of ASD (de Hoyos et al., 2024). They identified three independent genetic factors respectively underlying dimensions of language or cognition, behaviour, and motor development. These results replicate earlier twin findings that showed limited genetic overlap between different autistic trait domains (Robinson et al., 2012). More studies using genomic‐SEM in younger samples are needed to better understand the genetic architecture of other types of psychopathologies occurring during adolescence. Nonetheless, genomic‐SEM's capacity to model complex genetic structures and extend previous twin findings in younger populations is promising.

Taken together, these findings indicate widespread genetic overlap across psychopathologies in adolescence. They suggest that genetic factors associated with psychopathology tend to be broad and pleiotropic (i.e. genetic factors that are associated with many traits), although there is also evidence for (usually smaller) trait‐specific genetic effects. As such, genomic studies can gain power by widening the phenotype studied, though this does come at a cost in terms of specificity of findings as seen in adult depression (Cai, Choi, & Fried, 2020).

## Stability and change in adolescent psychopathology

In addition to providing useful insights on the co‐occurrence of psychopathologies, genetic studies have shed light on the processes underlying the developmental course of adolescent psychopathology. Whereas some youths experience persisting psychopathology over time, others have symptoms which fluctuate during adolescence (Murray, Eisner, Nagin, & Ribeaud, 2022; Oerlemans, Wardenaar, Raven, Hartman, & Ormel, 2020; Shore, Toumbourou, Lewis, & Kremer, 2018). Family and genomic studies have therefore examined how genetic and environmental influences contribute to the stability and change in psychopathology throughout adolescence (for reviews, see Akingbuwa et al., 2022; Hannigan, Walaker, Waszczuk, McAdams, & Eley, 2017).

### Stability in psychopathology

Twin studies show that genetic factors mostly account for the persistence in psychopathology during adolescence, including internalising, externalising, neurodevelopmental, and thought disorders, as well as the p factor (Abdellaoui et al., 2012; Allegrini et al., 2020; Chang, Lichtenstein, Asherson, & Larsson, 2013; Havers, Taylor, & Ronald, 2019; Martini et al., 2024; Waszczuk, Zavos, Gregory, & Eley, 2016; Wichers et al., 2013). Genetic influences that begin in early adolescence tend to persist into late adolescence, suggesting that genetic factors that emerge early in life may have enduring influences on psychopathology (Fairweather‐Schmidt & Wade, 2015; Hannigan, McAdams, & Eley, 2017; Hannigan, Walaker, et al., 2017; Lewis & Plomin, 2015; Martini et al., 2024). However, contributions from these persisting genetic influences progressively diminish with age, indicating that the magnitude of their influence may not be fixed across development.

Genomic studies support most of these findings by showing significant associations between polygenic scores and trajectories of persistent psychopathology in adolescence (Agnew‐Blais et al., 2021; Belsky et al., 2013; Havers, von Stumm, Cardno, Freeman, & Ronald, 2023; Kwong et al., 2019, 2021; Rice et al., 2019; Riglin et al., 2019; Shakeshaft et al., 2023; Weavers et al., 2021). Although longitudinal twin studies offer the possibility to examine whether etiological contributions persist or emerge over time through sophisticated structural equation models (e.g. Cholesky decomposition), the extent to which influences from common genetic variants persist or emerge throughout adolescence is still unclear. As genetic data in longitudinal cohorts and new genomic modelling techniques become available, it will become possible to examine these questions using individual genotypes.

Finally, longitudinal studies also suggest that chronic psychopathology may be more strongly influenced by genetic factors than sporadic or acute presentations. Notably, the heritability of psychopathology is typically higher when considering its stability over multiple time points rather than its level at individual time points (Cheesman et al., 2018; Funk, Morneau‐Vaillancourt, Palaiologou, & Eley, in preparation; Lubke et al., 2016; Waszczuk et al., 2014, 2016). For example, one study showed that extracting the stability in emotional symptoms from ages 7 to 16 years increased both twin and SNP heritability estimates from 45% to 76% and 5% to 15% respectively (Cheesman et al., 2018). Analysing the persistence of psychopathology across development is likely to help identify individual genetic variants, especially for disorders with lower heritability such as anxiety.

### Change in psychopathology

Studies have also integrated genetic methods into longitudinal approaches such as growth curve modelling to examine genetic influences on change in psychopathology over time. For the most part, twin studies show that change in psychopathology is accounted for by new genetic and environmental influences emerging over time (Fairweather‐Schmidt & Wade, 2015; Hannigan, McAdams, et al., 2017; Hannigan, Walaker, et al., 2017; Lewis & Plomin, 2015; Martini et al., 2024). This applies to different psychopathologies, including anxiety and depression (Gillespie, Eaves, Maes, & Silberg, 2015; Hatoum, Rhee, Corley, Hewitt, & Friedman, 2018; Ksinan & Vazsonyi, 2019), aggressiveness, delinquency, substance use, callous‐unemotional trait, psychopathic personality, and conduct problems (Hatoum et al., 2018; Pingault, Rijsdijk, Zheng, Plomin, & Viding, 2015; Takahashi, Pease, Pingault, & Viding, 2021; Tuvblad, Bezdjian, Raine, & Baker, 2013; Zellers et al., 2020), autistic traits (Martini et al., 2024), and psychopathic personality or psychotic experiences in adolescence (Zavos et al., 2016). Notably, change in ADHD symptoms may be an exception to this pattern. One study shows that change in hyperactivity/impulsivity may be explained by genetic influences, whereas change in inattention may be accounted for by environmental influences (Pingault, Viding, et al., 2015). Overall, twin studies are fairly consistent in showing that the genetic and environmental factors underlying fluctuations in psychopathology are dynamic rather than fixed across development.

Far fewer genomic studies have examined change in psychopathology during adolescence. In keeping with twin data, polygenic scores have been found to predict change in externalising problems (Bakken et al., 2024; Deak et al., 2022; Zaso, Maisto, Glatt, Hess, & Park, 2020). However, polygenic scores have not been found to impact change in anxiety and depressive symptoms in adolescence (Bakken et al., 2024; Nelemans et al., 2021). As such, more genomic studies are needed to clarify the role of common genetic variants in explaining fluctuations in psychopathology during adolescence.

Taken together, these new findings mostly confirm what earlier studies showed (Hannigan, McAdams, et al., 2017; Hannigan, Walaker, et al., 2017). First, the stability in psychopathology during adolescence is explained by genetic influences present early in adolescence that persist, though perhaps diminish in magnitude, over time. Second, change in psychopathology is explained by new genetic and environmental influences emerging over time. Adolescence is a period of rapid biological and social development, and studies show that this growth is reflected by dynamic etiological factors.

## Intergenerational transmission of psychopathology

Another area of genetic research that has progressed significantly in recent years is the intergenerational transmission of psychopathology. Decades of research indicate that psychopathology runs in families, and because biological family members share both genetic and environmental factors, different mechanisms can explain this intergenerational transmission (Foley et al., 2001; Lawrence, Murayama, & Creswell, 2019; McAdams et al., 2023). First are effects driven by genetic factors or direct genetic effects (Kong et al., 2018; Lynch & Walsh, 1998). Direct genetic effects refer to the process whereby children's psychopathology is influenced by genes transmitted by their parents. A second mechanism by which intergenerational transmission can occur is via environmental effects. These environmental effects can be driven by parents' genes influencing children indirectly through the environment they provide, a process called genetic nurture or indirect genetic effects (see Table 1). They can also be driven by other influences present in the environment and unrelated to the parent and the child's genetic factors (e.g. exposure to natural catastrophe). Recent studies have contributed to improve our understanding of these mechanisms (for a review, see Jami et al., 2021), but intergenerational research on psychopathology (especially in adolescence) is still relatively new and findings need to be interpreted with caution.

### Transmission of internalising disorders

Recent studies provide a complex picture of the mechanisms underlying the intergenerational transmission of internalising disorders. Some studies indicate that this transmission may operate via one route only, either genetic or environmental, while others suggest that multiple co‐occurring mechanisms are involved.

Several studies using the children‐of‐twins or other family‐based designs find no evidence of genetic transmission, with intergenerational resemblance arising solely from families living together (Burt, Clark, & Neiderhiser, 2022; Eley et al., 2015), in keeping with early findings from this field (Silberg, Maes, & Eaves, 2010; Singh et al., 2011). One explanation of such findings is that this transmission may occur via environmental mechanisms. For example, one study used a new genetically sensitive design including both biological and stepfathers from the US‐based Nonshared Environment in Adolescent Development study (Burt et al., 2022). This study shows that the association between fathers' depressive symptoms and adolescents' psychopathology may be environmentally mediated and explained via father‐child conflict rather than shared genetic factors. However, because the direction of this association remains unclear, both parent and child could influence one another in this association.

In contrast, other family and genomic studies show that the transmission of internalising disorders may operate via direct genetic effects (Grabow et al., 2017; Jami et al., 2023; Shakeshaft et al., 2024). One genomic study in the UK‐based Millenium Cohort Study found that polygenic scores computed from alleles that were transmitted from both parents were significantly associated with youth emotional problems from ages 3 to 17 years (Shakeshaft et al., 2024). These associations became stronger during adolescence, which could suggest that direct genetic effects on internalising disorders may become more important with age. However, because polygenic scores reflect genetic influences in adults (as GWAS were conducted in adult samples), it is also possible that the prediction by polygenic scores becomes larger as participants get closer to adulthood.

Finally, studies in the Norwegian Mother and Child Birth Cohort Study suggest that internalising disorders may be transmitted via more than one co‐occurring mechanism (Ahmadzadeh et al., 2023; Cheesman et al., 2020; Hannigan et al., 2018). For example, two studies using family‐based designs found that maternal depressive symptoms predicted child internalising problems through both direct genetic transmission and environmental mechanisms occurring beyond genetic factors shared by mother and child (Ahmadzadeh et al., 2023; Hannigan et al., 2018). These findings were corroborated by a genomic study showing that depressive symptoms in children were explained by both direct and indirect genetic transmission (Cheesman et al., 2020).

Collectively, family and genomic studies indicate that different mechanisms may account for the intergenerational transmission of internalising disorders. Some family studies point to environmental rather than genetic processes (Burt et al., 2022; Eley et al., 2015; Silberg et al., 2010; Singh et al., 2011) and others indicate that both direct genetic and environmental effects may also be involved (Ahmadzadeh et al., 2023; Hannigan et al., 2018). One study showed that these environmental effects may occur through indirect genetic effects, where parental genes affect the environment they provide, which then affect their child (Cheesman et al., 2020). However, research on the intergenerational transmission of internalising problems is still emerging and more studies are needed to clarify these results.

### Transmission of externalising disorders

Evidence regarding the transmission of externalising disorders is similarly inconsistent and also likely to be impacted by sample size and power issues. First, a few studies found evidence of both direct and indirect genetic effects on externalising disorders (Allegrini et al., 2023; Eilertsen et al., 2022; Kuo et al., 2022; Saunders, Liu, Vrieze, McGue, & Iacono, 2021; Thomas et al., 2023), in support of earlier children‐of‐twin studies (e.g. Silberg, Maes, & Eaves, 2012). For example, evidence from the US‐based Collaborative Studies on the Genetics of Alcoholism suggests that parental genotypes may be associated with adolescents' externalising and alcohol use through both direct and indirect genetic pathways (Kuo et al., 2022; Thomas et al., 2023). Interestingly, these studies found that parenting characteristics could be some of the intermediate pathways by which indirect genetic effects occur.

Second, a few studies provide evidence for direct, but not for indirect genetic effects on externalising disorders (Frach et al., 2024; Kretschmer, Vrijen, Nolte, Wertz, & Hartman, 2022; Tanksley et al., 2023; van der Laan et al., 2023). One longitudinal study examined how a polygenic score for externalising disorders related to child and adolescent externalising problems in two UK‐based cohorts, the Environmental Risk Longitudinal Twin Study and Millenium Cohort Study (Tanksley et al., 2023). Results show that polygenic score effects observed within families were similar to those found in the population, suggesting direct rather than indirect genetic effects, as within‐family analyses control for indirect genetic effects (since the effect of parental genotypes on the family environment is the same across siblings). Interestingly, this study also found that direct genetic effects were stronger in childhood than in adolescence. These findings contrast with those from Shakeshaft et al. (2024), who showed that direct genetic effects on internalising disorders could *increase* with age. These longitudinal findings need to be replicated and interpreted with caution, but this illustrates how intergenerational mechanisms may differ across different types of psychopathologies.

Overall, recent intergenerational studies show that externalising disorders are likely to be explained by direct genetic effects (Allegrini et al., 2023; Eilertsen et al., 2022; Frach et al., 2024; Kretschmer et al., 2022; Kuo et al., 2022; Saunders et al., 2021; Tanksley et al., 2023). Some studies also suggest that parental genes may affect children through the environment they provide, that is, indirect genetic effects (Allegrini et al., 2023; Eilertsen et al., 2022; Kuo et al., 2022; Saunders et al., 2021; Thomas et al., 2023). Again, more studies are needed to clarify the mechanisms underlying the intergenerational transmission of externalising disorders.

### Transmission of neurodevelopmental disorders, thought disorders, and p factor

In addition to internalising and externalising disorders, recent studies provide insights into the transmission of ADHD, thought disorders, and the p factor, phenotypes rarely examined by previous intergenerational family studies (McAdams et al., 2014). In the past 2 years, several intergenerational studies on ADHD have been published. Most studies find that only direct genetic effects account for childhood ADHD (Allegrini et al., 2023; Eilertsen et al., 2022; Jami et al., 2023; Kleppesto et al., 2024; Martin et al., 2023; Pingault et al., 2023; Voronin et al., 2024; Wechsler et al., 2022). However, two studies also suggest that environmental (Kleppesto et al., 2024), and more specifically indirect genetic effects (Allegrini et al., 2023), may also account for the transmission of ADHD, but to a lesser extent than direct genetic effects (Kleppesto et al., 2024). One further study did not find any evidence of direct or indirect genetic effects on ADHD using polygenic scores (Axelrud et al., 2023). As such, recent studies on the intergenerational transmission of ADHD mostly point to direct genetic effects, with some evidence that environmental mechanisms may contribute to ADHD. The fact that these findings are mostly consistent could be explained by the higher heritability of ADHD and thus stronger statistical power to detect genetic effects (74% for ADHD vs. 40%–60% for internalising and externalising disorders; Faraone & Larsson, 2019; Polderman et al., 2015).

Recent studies have also examined intergenerational mechanisms underlying psychotic experiences and the p factor. One study examined the associations between parent polygenic scores and offspring psychotic symptoms in the Brazilian High Risk Cohort study (Axelrud et al., 2023). Findings show no evidence of either direct or indirect genetic effects. Another study examined the intergenerational transmission of risk for a general p factor in the Norwegian Mother and Child Birth Cohort Study (Allegrini et al., 2023). Findings suggest that children's p was influenced by both direct and indirect genetic effects. More research is needed to clarify the transmission mechanisms underlying these phenotypes, but these studies nonetheless provide interesting preliminary evidence.

In conclusion, research on the intergenerational transmission of psychopathology has made significant progress in recent years. Whereas findings on internalising and externalising disorders often point to different co‐occurring genetic and environmental mechanisms of transmission, those on ADHD mostly indicate that direct genetic effects may be involved. Researchers are also investigating the transmission of other phenotypes that had not been studied yet, including thought disorders and the p factor. However, these findings need to be interpreted carefully as existing intergenerational studies may lack statistical power to detect genetic effects or may be limited by the narrow predictive capacity of polygenic scores.

## Gene–environment interplay in adolescent psychopathology

Research shows that genetic and environmental factors often work together in shaping risk for psychopathology. Two key forms of gene–environment interplay are described in the literature (Lau & Eley, 2004; Rutter, Moffitt, & Caspi, 2006). Gene–environment correlation (rGE) occurs when genetic factors affect the likelihood of experiencing certain environments (Plomin, 2014), whereas gene–environment interaction (GxE) refers to the process by which the effect of the environment depends on genetic factors or vice versa (Dick, 2011).

### Gene–environment correlations

Twin research shows that the vast majority of environmental factors, including stressful life events, parenting behaviours, the family environment and social interactions, are under genetic influences. As such, genetic and environmental factors are not independent of one another (Kendler & Baker, 2007). Three processes have been described that lead to rGE (Plomin, DeFries, & Loehlin, 1977; Scarr & McCartney, 1983). First is passive rGE, where parents' genes influence the environment they provide, which in turn affects their children. This is similar to indirect genetic effects or genetic nurture. Second is evocative or reactive rGE, where individuals' genetic predispositions evoke a response in others. Finally, in active rGE, individuals actively select their environment based on their genetic predispositions.

Twin studies from the past decade largely replicate earlier findings and demonstrate significant rGE between most psychopathologies and adverse environmental variables in adolescence (Mann et al., 2016; McAdams, Gregory, & Eley, 2013; Samek et al., 2015; Shakoor et al., 2016; Törn et al., 2015; Waldman et al., 2016). Interestingly, evidence is emerging that psychological processes such as environmental sensitivity (how responsive people are to the world around them) and anxiety sensitivity (how responsive people are to anxiety‐related internal and external cues) are influenced by the same genetic factors as life events (Peel et al., 2023). As such, it is possible that these responsivity/sensitivity traits may mediate genetic influences on life events (for a practitioner review on responsivity to the environment, see Assary, Krebs, & Eley, 2023). Recent genomic studies also generally show significant rGE between psychopathology polygenic scores and several environmental exposures. Examples include associations between polygenic scores for ASD, depression, post‐traumatic stress disorder, risky behaviours, schizophrenia, substance use and environmental exposures including parenting behaviours, family dysfunction, peer substance use and environmental adversity (Ensink et al., 2020; Krapohl et al., 2016; Ksinan, Smith, Barr, & Vazsonyi, 2022; Kuo et al., 2021; Newbury et al., 2022; Pasman et al., 2023; Peel et al., 2022). These findings collectively suggest a widespread network of rGE involving numerous environmental variables.

To account for these widespread rGE, researchers have recently adopted a broader analytical approach than previous studies that focused on specific environmental factors. One study examined the interplay between the genome and the exposome, defined as the totality of environmental exposure – in this case 133 factors related to the family, peers, school, neighbourhood, life events and broader environment (Choi et al., 2022). Findings showed that influences from common genetic variants on youth externalising symptoms were significantly attenuated (from 19% to 8% of variance explained) after accounting for the exposome, suggesting that environmental exposures may be an intermediate pathway by which genetic factors predict externalising problems (Choi et al., 2022). However, genetic influences on internalising symptoms remained unchanged (at 6%) after accounting for the exposome, suggesting that rGE may be more prominent for externalising than internalising problems. This finding is also consistent with twin studies showing that externalising behaviour is one of the few traits showing significant shared environmental influences, which can reflect passive rGE (Neiderhiser et al., 2004; Polderman et al., 2015). This new approach combining the genome and the exposome is promising as it may help understand how the environment as a whole contributes to psychopathology.

Finally, longitudinal studies have examined how rGE may change across development. Family studies suggest that passive rGE may be more present earlier in adolescence, whereas evocative or active rGE may be greater later in adolescence (Hannigan, McAdams et al., 2017; Marceau et al., 2016). These findings suggest that parents may have a larger impact earlier in life given that adolescents typically increasingly select their environment as they grow up. However, these developmental changes in rGE have not been consistently supported by genomic studies, and there is no robust evidence that associations between polygenic scores and environmental exposures increase during adolescence (Machlitt‐Northen, Keers, Munroe, Howard, & Pluess, 2022; Newbury et al., 2022).

### Gene–environment interactions

As for rGE, researchers have proposed different processes by which GxE can occur, including the diathesis‐stress, bioecological, vantage sensitivity and differential susceptibility models (for more details, see Belsky & Pluess, 2009; Monroe & Simons, 1991; Pluess, 2015; Pluess & Belsky, 2013). Research on GxE contributing to adolescent psychopathology is still in its infancy and has mostly focused on examining negative rather than positive environmental exposures. Many genomic studies have examined GxE using candidate genes (Assary, Vincent, Keers, & Pluess, 2018), but we do not cover them as this approach is ill‐suited to the study of polygenic psychopathological traits (Andreassen et al., 2023).

Twin studies on GxE in adolescence have mostly focused on internalising and externalising disorders (for reviews, see Burt, 2015; Neiderhiser & Chen, 2023). Earlier studies show inconsistent findings, with some providing significant evidence and others not. The most consistent finding appears to be that genetic influences on adolescent substance use tend to be larger in contexts supporting substance use, supporting the diathesis‐stress model (Dick et al., 2015; Neiderhiser & Chen, 2023). Recent studies suggest significant GxE where adverse family conditions or neighbourhoods may modulate environmental or genetic influences on internalising and externalising problems in adolescence (Dash et al., 2023; Nikstat, Beam, & Riemann, 2023; Wright & Schwartz, 2021). However, these findings were not always replicated (Burt, Clark, Pearson, Klump, & Neiderhiser, 2020). As such, current twin findings are still insufficient to conclude that GxE are involved in adolescent internalising and externalising disorders.

Likewise, genomic studies do not provide consistent evidence of GxE (Lewis & Vassos, 2020). Studies in adults show inconclusive findings regarding GxE between polygenic risk for depression and trauma (Coleman et al., 2020; Peyrot et al., 2018), polygenic risk for schizophrenia and early childhood adversity (Woolway et al., 2022), and polygenic risk for different traits and substance use (Pasman, Verweij, & Vink, 2019). Genomic studies in adolescence present a similar pattern of inconsistent findings. Some studies show no robust evidence of GxE involving polygenic scores and adolescent psychopathology (Elkrief et al., 2023; He & Li, 2022; Kandaswamy et al., 2022; Ksinan et al., 2022; Misztal, Tio, Mohan, & Felsky, 2023; Mooney et al., 2023; Østergaard et al., 2020; Perret et al., 2023; Plomin, Gidziela, Malanchini, & Von Stumm, 2022). In contrast, other studies observed significant GxE, where a greater polygenic liability for psychopathology led to poorer outcomes in contexts of negative environmental exposures, supporting the diathesis‐stress model (Bares et al., 2020; Joo et al., 2022; Misztal et al., 2023; Nelemans et al., 2021). Notably, Choi et al. (2022) showed that GxE involving the exposome respectively explained 13% and 33% of the variance in internalising and externalising problems in young adolescents. This suggests that considering a wider range of environmental exposures may be key in detecting GxE contributions to adolescent psychopathology. Therefore, genomic research on GxE provides inconclusive findings, although the integration of the genome and the exposome into GxE models may constitute promising research avenues.

In summary, research on gene–environment interplay shows that genetic and environmental factors are not independent and that their effects are conditional on one another. These results also need to be interpreted taking into consideration that GxE may be biassed in the presence of unobserved rGE (Purcell, 2002; Rathouz, van Hulle, Rodgers, Waldman, & Lahey, 2008; van der Sluis, Posthuma, & Dolan, 2012). For example, both GxE and rGE processes could explain why individuals show greater genetic influences in contexts of environmental adversity (Purcell, 2002). Methodologically, GxE and rGE are difficult to tease apart, and studies are often limited by this potential source of bias. Researchers are actively working on this issue and proposing new methods such as the multiple‐children‐of‐twins design to study GxE in the presence of rGE (e.g. Badini et al., 2024). More longitudinal studies are needed to elucidate the exact mechanisms by which these processes unfold during adolescence, especially at the genomic level.

## Psychological treatment outcomes

In recent years, researchers have become increasingly interested in understanding how genetic predispositions may influence the way individuals respond to psychological treatment, a form of GxE also referred to as therapygenetics (Eley et al., 2012; Lester & Eley, 2013). There is a high level of variability in how individuals respond to psychological treatment (Skelton et al., 2023). In young people, as in adults, only half of those treated with cognitive behavioural therapy remit (James, Reardon, Soler, James, & Creswell, 2020). Genetic predispositions could be one factor explaining why adolescents show different responses to psychological treatment.

The sparse findings on therapygenetics largely come from studies of adults and children rather than adolescents, as well as from genomic rather than twin studies, and available findings are inconsistent. Previous GWAS on treatment response have been limited by small sample sizes, as recruiting individuals who experience treatment is challenging (Coleman et al., 2016; Rayner et al., 2019). In addition, a recent review concluded that the utility of polygenic scores in predicting treatment outcomes for psychosis, bipolar disorder, and major depressive disorder in adults is limited (Fusar‐Poli, Rutten, van Os, Aguglia, & Guloksuz, 2022).

However, other findings may be more promising. For example, one study shows that polygenic scores may significantly improve the prediction of depression remission, when considered in combination with 45 other demographic and clinical factors (Wallert et al., 2022). Another study of adolescents suggests that a polygenic score for environmental sensitivity may partially account for psychological treatment response (Keers et al., 2016). Results showed that children and adolescents who were genetically predisposed to be sensitive to the environment benefited more from one‐to‐one delivery of psychological treatment as compared to group treatment or parent‐led treatment.

As such, current knowledge on therapygenetics is limited by a lack of family studies and insufficient statistical power in genomic studies. Some findings on the role of genetic factors in psychological treatment response are promising, but more studies are needed to better understand the role of genetic factors in predicting adolescent treatment outcomes. However, it remains plausible that genetic risk will be useful alongside more traditional clinical and demographic variables when trying to predict likely outcome following psychological treatment.

## Conclusions: Remaining gaps and future directions

Despite these recent advances, we note three remaining gaps that need to be addressed in future work. First, genomic studies have for the most part been conducted in participants of European ancestry and in Western, Educated, Industrialised, Rich and Democratic (WEIRD) societies. It is crucial to increase inclusivity and diversity in genetic research so that the benefits of research can be generalised to all (Fatumo et al., 2022).

Second, studies on internalising disorders often show more inconsistent findings than those on other psychopathologies. One explanation could be that internalising disorders tend to be less heritable than externalising, neurodevelopmental or thought disorders (Polderman et al., 2015). It could also be that there is a high level of heterogeneity in symptom manifestation, which could complicate accurate phenotypic assessment. As rates of anxiety and depression are currently rising in adolescents (Dykxhoorn et al., 2024), there is a pressing need for more studies focusing on adolescent internalising disorders.

Third, most genomic findings come from cross‐sectional studies and focus on childhood rather than adolescence. More longitudinal studies covering adolescence would help clarify whether genetic variants show enduring influences on psychopathology or whether new genetic variants emerge during adolescence. It would also contribute to a better understanding of the intergenerational transmission mechanisms underlying adolescent psychopathology (especially for ASD, as we know little about the transmission of this highly heritable phenotype). More longitudinal studies are also needed to improve our comprehension of gene–environment interplay. Twin studies suggest that rGE processes change across development, but this finding has not yet been replicated in genomic studies. In addition, it remains largely unclear whether GxE processes predict individual differences in adolescent psychopathology and if so, how these processes unfold across adolescence. Finally, the literature on therapygenetics is limited, and more studies—both family and genomic—are needed to shed light on the role of genetic factors in psychological treatment in adolescents.

In the past decade, considerable progress has been achieved in research on the genetics of adolescent psychopathology. We now have a better understanding of how genetic influences overlap across different traits, unfold over time, are transmitted from one generation to the next, and work together with the environment to shape risk for psychopathology. This is an exciting time for researchers in behavioural genetics, as new genomic approaches, especially those combining family‐based and genetic data, will provide new research possibilities in the near future. In time, it is likely that this information will become useful in understanding presentation, course and even outcome in adolescent mental health.

**Key points**

- Psychopathology is common and often begins during adolescence. Genetic factors account for around 50% of individual differences in psychopathology in adolescents.
- Recent family and genomic studies provide crucial insights into the mechanisms—both genetic and environmental—underlying the onset, persistence, and remission of psychopathology in adolescence.
- Genetic factors play a role in explaining the (1) co‐occurrence between psychopathologies, (2) their stability and change over time, (3) the mechanisms underlying their intergenerational transmission, (4) the extent to which they work together with the environment to predict psychopathology, and (5) psychological treatment outcomes.
- Future research will need to address the lack of diversity and longitudinal designs in genomic studies, as well as the fact that findings on internalising disorders are often inconsistent.

## Data Availability

This paper is a review and does not involve the analysis of primary data.
